# Supplementary material for: MetaRibo-Seq measures translation in microbiomes
Source: Nat Commun. 2020 Jun 29;11:3268. doi: 10.1038/s41467-020-17081-z (PMC7324362; doi:10.1038/s41467-020-17081-z)
Supplement: Supplementary file 10 — Supplementary Data 7 [file 41467_2020_17081_MOESM10_ESM.zip › File2/Confidence_High_Taxonomy/290595_out.krona.html]

Javascript must be enabled to view this page.

members
magnitude
magnitudeUnassigned
count
unassigned
taxon
rank

290595\_out

308

308
2
superkingdom

308
976
phylum

200643
class
308

308
order
171549

308
815
family

genus
816

SRS021219\_contig\_number\_7200SRS104636\_contig\_number\_4330
2
308

species
28116

SRS015065\_contig\_number\_23842SRS019693\_contig\_number\_1851SRS046369\_contig\_number\_11020SRS050026\_contig\_number\_3355SRS053649\_contig\_number\_3262SRS054956\_contig\_number\_1733SRS062701\_contig\_number\_2442
7

2292928
species

SRS019267\_contig\_number\_466SRS077194\_contig\_number\_21745SRS143598\_contig\_number\_2241SRS144506\_contig\_number\_20782SRS147557\_contig\_number\_contig-100\_362.182552SRS893230\_contig\_number\_2308
6


SRS011134\_contig\_number\_contig-100\_814.306651SRS011239\_contig\_number\_3704SRS011239\_contig\_number\_18217SRS011302\_contig\_number\_30893SRS011405\_contig\_number\_9889SRS012273\_contig\_number\_21806SRS012969\_contig\_number\_41032SRS013158\_contig\_number\_2596SRS013638\_contig\_number\_1397SRS013687\_contig\_number\_1930SRS013800\_contig\_number\_1945SRS013940\_contig\_number\_20827SRS013951\_contig\_number\_6157SRS014235\_contig\_number\_1974SRS014287\_contig\_number\_16000SRS014313\_contig\_number\_870SRS014412\_contig\_number\_13288SRS014683\_contig\_number\_12451SRS014923\_contig\_number\_35017SRS015190\_contig\_number\_10488SRS015264\_contig\_number\_18047SRS015431\_contig\_number\_43501SRS015578\_contig\_number\_2466SRS015663\_contig\_number\_29323SRS015694\_contig\_number\_22643SRS015782\_contig\_number\_34977SRS015794\_contig\_number\_7074SRS015960\_contig\_number\_10808SRS016018\_contig\_number\_5916SRS016132\_contig\_number\_1088SRS016203\_contig\_number\_1324SRS016753\_contig\_number\_2578SRS016840\_contig\_number\_383SRS016954\_contig\_number\_19739SRS017103\_contig\_number\_contig-100\_1656.209257SRS017247\_contig\_number\_2246SRS017521\_contig\_number\_43039SRS017745\_contig\_number\_1022SRS017821\_contig\_number\_17255SRS017859\_contig\_number\_1028SRS018133\_contig\_number\_1729SRS018351\_contig\_number\_2077SRS018427\_contig\_number\_32921SRS018541\_contig\_number\_9893SRS018575\_contig\_number\_8404SRS018623\_contig\_number\_17439SRS018836\_contig\_number\_8577SRS018888\_contig\_number\_739SRS018936\_contig\_number\_96SRS019068\_contig\_number\_55361SRS019161\_contig\_number\_3574SRS019286\_contig\_number\_1791SRS019397\_contig\_number\_1537SRS019445\_contig\_number\_21536SRS019496\_contig\_number\_968SRS019601\_contig\_number\_26230SRS019685\_contig\_number\_7056SRS019808\_contig\_number\_11645SRS020328\_contig\_number\_20020SRS020394\_contig\_number\_6358SRS020622\_contig\_number\_7590SRS021948\_contig\_number\_26740SRS022071\_contig\_number\_14263SRS022137\_contig\_number\_2310SRS022524\_contig\_number\_8279SRS022609\_contig\_number\_37597SRS023715\_contig\_number\_23361SRS023971\_contig\_number\_4523SRS024009\_contig\_number\_5213SRS024075\_contig\_number\_1030SRS024132\_contig\_number\_34068SRS024132\_contig\_number\_39001SRS024663\_contig\_number\_10631SRS042284\_contig\_number\_1087SRS042628\_contig\_number\_15190SRS043411\_contig\_number\_11342SRS043768\_contig\_number\_23930SRS045004\_contig\_number\_6075SRS045645\_contig\_number\_8981SRS045739\_contig\_number\_854SRS045826\_contig\_number\_2817SRS046068\_contig\_number\_281SRS046717\_contig\_number\_7667SRS047433\_contig\_number\_12606SRS048060\_contig\_number\_9446SRS048870\_contig\_number\_18720SRS048981\_contig\_number\_3228SRS049402\_contig\_number\_8974SRS049446\_contig\_number\_1806SRS049773\_contig\_number\_28286SRS049896\_contig\_number\_6724SRS049995\_contig\_number\_37731SRS050299\_contig\_number\_10596SRS050520\_contig\_number\_20SRS050752\_contig\_number\_23515SRS050925\_contig\_number\_14024SRS050941\_contig\_number\_2461SRS050974\_contig\_number\_contig-100\_18895.18896SRS050998\_contig\_number\_14069SRS051031\_contig\_number\_16533SRS051610\_contig\_number\_8961SRS052027\_contig\_number\_20635SRS052697\_contig\_number\_32300SRS053087\_contig\_number\_2508SRS053335\_contig\_number\_11716SRS054905\_contig\_number\_5974SRS055017\_contig\_number\_2600SRS055982\_contig\_number\_3394SRS056259\_contig\_number\_12052SRS056273\_contig\_number\_20668SRS056537\_contig\_number\_11002SRS057478\_contig\_number\_2744SRS058723\_contig\_number\_8081SRS058770\_contig\_number\_21748SRS062427\_contig\_number\_4260SRS063190\_contig\_number\_10622SRS063370\_contig\_number\_9671SRS063489\_contig\_number\_1402SRS063489\_contig\_number\_2764SRS063518\_contig\_number\_20573SRS063928\_contig\_number\_100SRS064232\_contig\_number\_4205SRS064276\_contig\_number\_38728SRS064645\_contig\_number\_80SRS064757\_contig\_number\_7118SRS065176\_contig\_number\_9055SRS065504\_contig\_number\_3619SRS074670\_contig\_number\_2830SRS075021\_contig\_number\_2910SRS075078\_contig\_number\_10629SRS075716\_contig\_number\_1280SRS075773\_contig\_number\_15338SRS075821\_contig\_number\_3738SRS075878\_contig\_number\_4782SRS075984\_contig\_number\_5164SRS076929\_contig\_number\_24201SRS076976\_contig\_number\_2157SRS077086\_contig\_number\_12046SRS077194\_contig\_number\_contig-100\_488.192508SRS077294\_contig\_number\_3512SRS077335\_contig\_number\_2981SRS077392\_contig\_number\_27551SRS077454\_contig\_number\_15682SRS077502\_contig\_number\_5831SRS077552\_contig\_number\_13146SRS077589\_contig\_number\_contig-100\_1133.50418SRS077589\_contig\_number\_contig-100\_74.53482SRS077849\_contig\_number\_28817SRS078176\_contig\_number\_17256SRS078419\_contig\_number\_21079SRS097920\_contig\_number\_24143SRS097958\_contig\_number\_23799SRS098073\_contig\_number\_12316SRS098571\_contig\_number\_26840SRS098655\_contig\_number\_3822SRS100021\_contig\_number\_887SRS101376\_contig\_number\_22721SRS101433\_contig\_number\_7612SRS104084\_contig\_number\_4133SRS1041031\_contig\_number\_8913SRS1041032\_contig\_number\_contig-100\_463.94319SRS1041036\_contig\_number\_4189SRS1041037\_contig\_number\_4542SRS1041095\_contig\_number\_93SRS1041112\_contig\_number\_2235SRS1041116\_contig\_number\_7574SRS1041118\_contig\_number\_4204SRS1041129\_contig\_number\_6150SRS1041130\_contig\_number\_2298SRS1041133\_contig\_number\_19118SRS1041140\_contig\_number\_10832SRS1041141\_contig\_number\_14285SRS1041144\_contig\_number\_1093SRS1041145\_contig\_number\_36316SRS1041147\_contig\_number\_663SRS1041157\_contig\_number\_4381SRS1041164\_contig\_number\_93SRS104327\_contig\_number\_704SRS104693\_contig\_number\_7109SRS104975\_contig\_number\_6541SRS1054691\_contig\_number\_11349SRS1054716\_contig\_number\_10545SRS1055049\_contig\_number\_3709SRS1055067\_contig\_number\_10564SRS1055076\_contig\_number\_12943SRS1055083\_contig\_number\_2504SRS1055095\_contig\_number\_147SRS140492\_contig\_number\_6574SRS140513\_contig\_number\_621SRS142503\_contig\_number\_19073SRS142542\_contig\_number\_11492SRS142599\_contig\_number\_27976SRS142781\_contig\_number\_8809SRS142923\_contig\_number\_928SRS143085\_contig\_number\_2886SRS143181\_contig\_number\_2994SRS143342\_contig\_number\_19782SRS143372\_contig\_number\_6806SRS143466\_contig\_number\_25981SRS143523\_contig\_number\_3299SRS143722\_contig\_number\_499SRS143780\_contig\_number\_5471SRS143991\_contig\_number\_17101SRS144135\_contig\_number\_12304SRS144183\_contig\_number\_1697SRS144297\_contig\_number\_12808SRS144362\_contig\_number\_32732SRS144537\_contig\_number\_contig-100\_1600.268096SRS144603\_contig\_number\_contig-100\_50.88895SRS144714\_contig\_number\_25459SRS145497\_contig\_number\_11743SRS146812\_contig\_number\_49882SRS147271\_contig\_number\_33194SRS147346\_contig\_number\_10048SRS147614\_contig\_number\_1861SRS147653\_contig\_number\_1438SRS147766\_contig\_number\_12531SRS147919\_contig\_number\_4058SRS147977\_contig\_number\_3221SRS148091\_contig\_number\_165SRS148319\_contig\_number\_3415SRS148721\_contig\_number\_7659SRS148817\_contig\_number\_598SRS148970\_contig\_number\_1605SRS149075\_contig\_number\_10582SRS149325\_contig\_number\_15740SRS150029\_contig\_number\_contig-100\_313.121462SRS892882\_contig\_number\_11050SRS893172\_contig\_number\_10634SRS893187\_contig\_number\_contig-100\_175.103950SRS893231\_contig\_number\_781SRS893253\_contig\_number\_81SRS893256\_contig\_number\_731SRS893270\_contig\_number\_19016SRS893279\_contig\_number\_13737SRS893292\_contig\_number\_contig-100\_967.49390SRS893358\_contig\_number\_9758SRS893366\_contig\_number\_4592SRS893373\_contig\_number\_12343SRS893378\_contig\_number\_4456SRS893382\_contig\_number\_2548SRS971276\_contig\_number\_33701
242
species
820

1

SRS024331\_contig\_number\_25232
387090
species

species
1946089

SRS076756\_contig\_number\_9899SRS077231\_contig\_number\_20589SRS1055022\_contig\_number\_18389SRS1055043\_contig\_number\_12049SRS1055099\_contig\_number\_10772SRS893295\_contig\_number\_11206
6

1

SRS021153\_contig\_number\_59
species
818

1

SRS024492\_contig\_number\_3767
162156
species

93975
species

SRS1041091\_contig\_number\_21700SRS1041134\_contig\_number\_12036SRS1041138\_contig\_number\_5001
3

species
47678
5

SRS019030\_contig\_number\_11838SRS023526\_contig\_number\_19574SRS024625\_contig\_number\_11642SRS045244\_contig\_number\_200SRS104400\_contig\_number\_22596

species
821
2

SRS014855\_contig\_number\_11181SRS098061\_contig\_number\_1544

species
556259
1

SRS097889\_contig\_number\_26768


SRS015854\_contig\_number\_3385SRS016056\_contig\_number\_54SRS017433\_contig\_number\_17324SRS022713\_contig\_number\_1084SRS101376\_contig\_number\_11388SRS1055069\_contig\_number\_7868SRS143148\_contig\_number\_6146SRS148253\_contig\_number\_1141SRS149181\_contig\_number\_23494
9
species
46506

species
457393
1

SRS043001\_contig\_number\_1488


SRS893342\_contig\_number\_900
1
species
817

species
585543

SRS011084\_contig\_number\_12812SRS014948\_contig\_number\_23180SRS015890\_contig\_number\_13183SRS016335\_contig\_number\_14454SRS016897\_contig\_number\_69SRS018049\_contig\_number\_contig-100\_13.14SRS018984\_contig\_number\_3447SRS019968\_contig\_number\_9208SRS021484\_contig\_number\_14023SRS047014\_contig\_number\_37554SRS050422\_contig\_number\_37367SRS052078\_contig\_number\_1791SRS076804\_contig\_number\_13153SRS077024\_contig\_number\_5191SRS077127\_contig\_number\_contig-100\_2323.110504SRS101268\_contig\_number\_13SRS104311\_contig\_number\_30993SRS142890\_contig\_number\_33935SRS149784\_contig\_number\_24606SRS893380\_contig\_number\_2723
20
